# Variation in Chin and Mandibular Symphysis Size and Shape in Males and Females: A CT-Based Study

**DOI:** 10.3390/ijerph17124249

**Published:** 2020-06-14

**Authors:** Tatiana Sella Tunis, Israel Hershkovitz, Hila May, Alexander Dan Vardimon, Rachel Sarig, Nir Shpack

**Affiliations:** 1 Department of Anatomy and Anthropology, Sackler Faculty of Medicine, Tel Aviv University, Ramat Aviv 69978, Israel; anatom2@tauex.tau.ac.il (I.H.); 2hilamay@gmail.com (H.M.); 2 Dan David Center for Human Evolution and Biohistory Research, Shmunis Family Anthropology Institute, Sackler Faculty of Medicine, Tel Aviv University, Ramat Aviv 69978, Israel; rachel.sarig@gmail.com; 3 Department of Orthodontics, The Maurice and Gabriela Goldschleger School of Dental Medicine, Sackler Faculty of Medicine, Tel Aviv University, Ramat Aviv 69978, Israel; andyva@post.tau.ac.il (A.D.V.); nir@shpack.co.il (N.S.); 4 Department of Oral Biology, The Maurice and Gabriela Goldschleger School of Dental Medicine, Sackler Faculty of Medicine, Tel Aviv University, Ramat Aviv 69978, Israel

**Keywords:** chin, mandibular symphysis, morphometrics, sexual dimorphism, facial attractiveness, computed tomography

## Abstract

The chin is a unique anatomical landmark of modern humans. Its size and shape play an important role from the esthetic perspective. However, disagreement exists in the dental and anthropological literature regarding the sex differences in chin and symphysis morphometrics. The “sexual selection” theory is presented as a possible reason for chin formation in our species; however, many other contradictory theories also exist. This study’s aims were therefore to determine how chin and symphysis size and shape vary with sex, and to discuss “sexual selection” theory as a reason for its formation. Head and neck computed tomography (CT) scans of 419 adults were utilized to measure chin and symphysis sizes and shapes. The chin and symphysis measures were compared between the sexes using an independent-samples *t*-test, a Mann–Whitney test, and the F-statistic. The chin width was significantly greater in males than in females (*p* < 0.001), whereas the chin height, area, and size index were significantly greater in females (*p* < 0.001). Symphysis measures did not differ significantly between the sexes. Size accounted for 2–14% of the chin variance and between 24–33% of the symphysis variance. Overall, the chin was found to be a more heterogeneous anatomical structure than the symphysis, as well as more sexually dimorphic.

## 1. Introduction

**Figure 1:**
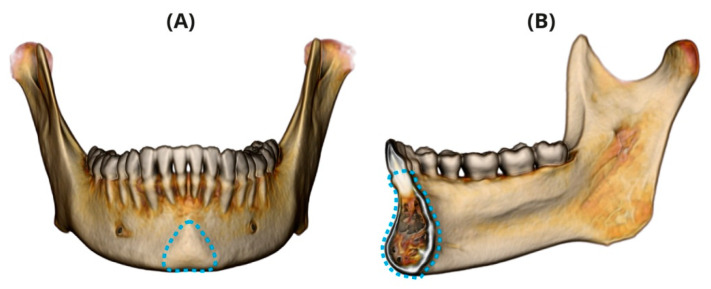
(**A**) Chin (mentum osseum). Frontal view of the mandible (using the volume rendering technique); chin borders (mental protuberance and bilateral mental tubercles) are denoted by a dotted line. (**B**) Mandibular symphysis. Midsagittal section; symphysis borders are denoted by dotted lines.

The chin (mentum osseum) is a distinctive feature of the anterior mandibular symphysis found only in our species, *Homo sapiens* [1]. It is characterized by a mental protuberance, a raised vertical structure that lies along the symphyseal midline, along with paired bulbous formations placed on each lateral side of its inferior margin (mental tubercles) (Figure 1A). The presence of a chin is already noticeable in the fifth fetal month [1,2] and the mandible retains this characteristic into adult life [3].

The mandibular symphysis isthe region where the two halves of the human fetal mandibular corpus are fused [4] (Figure 1B). As opposed to the chin, the mandibular symphysis is found in all hominoid mandibles and its morphology was functionally linked [5].

Several hypotheses have been raised over the years to elucidate the reason for the chin’s appearance in humankind. The “sexual selection” hypothesis is among the most common; it states that the appearance of the chin in humans and its size and shape variation are due to sexual selection, relating to facial attractiveness [6,7,8,9].

In modern dentistry, the chin and symphysis size and shape play an important role from the esthetic perspective [10]. Chin height and width are considered important factors in perceiving facial attractiveness. Deformities in chin height (when greater than 50% and 58% of the lower anterior facial height in males and females, respectively) were found to be the least attractive facial characteristic, and consequently, surgery was required to correct the facial appearance [11]. A squared contour of the chin in females is considered unattractive since it gives the face a masculine appearance and thus diminishes the appearance of femininity [8]. Narrowing genioplasty to achieve a feminine and slim lower face is a well-documented procedure that is practiced to improve the facial appearance and the lower facial contour [12,13]. Valenzano et al. [14] applied morphometric analysis(quantitative analysis of the chin and symphysis size and shape) regarding facial attractiveness. However, they failed to find an association between attractiveness and sexual dimorphism of the lower face (including the chin). 

Qualitative differences in the chin shape between the sexes are well known in anthropology: males possess broad and prominent chins with a more square appearance and females possess pointed chins [15,16,17,18,19]. Thayer and Dobson [9] compared the chin shape between the sexes using elliptical Fourier function analysis (EFFA) and found that sexual dimorphism exists in its shape. However, there was a high degree of overlap in the chin shape between males and females due to a large degree of variation in each sex. Nevertheless, males possessed a taller symphysis, a more protrusive mentum osseum, and larger tubercula laterale than females did. Garvin and Ruff [17] used 3D surface laser scans to show that the absolute surface area and the volume of the chin are sexually dimorphic; however, the sex differences became non-significant when they were standardized for size. Garvin and Ruff’s morphometric shape analysis revealed a relatively high overlap in the chin shape between the sexes. Males displayed relatively more prominent mental protruberances, lateral tubercles, and taller chins than did females. Coquerelleet al. [20] and Franklin et al. [21,22] used the geometric morphometric method to find that the sexual dimorphism of the chin and mandibular symphysis is age-dependent. According to Coquerelleet al. [20], there is amarked sexual dimorphism in symphyseal shape at birth. It dramatically decreases between the ages of 4–14 years, and increases again afterward. Adult dimorphism was similar to that of the early postnatal stages;namely, males had a square-shaped chin compared with a round-shaped chin in females. However, Daegling [23] and Pampush [24] did not find any differences in chin thickness, size, and shape between the sexes. Daegling showed that a substantial overlap of the chin shape exists between the sexes in the midsagittal section. 

Since the chin and symphysis are major components of the lower third of the face, it is essential to determine whether their size and shape are sex-dependent for clinical and anthropological purposes. Whereas some studies clearly showed the existence of sexual dimorphism in the chin and symphysis [9,17], others did not conclude that it exists [23,24]. The inconsistent findings reported in the dental and anthropological literature may be related to the different methodologies used to quantify the chin and symphysis, and to the lack of age and size control; hence, the appropriateness of the “sexual selection” hypothesis is questionable.

This study aimed to compare the magnitude of sexual dimorphism in the chin and symphysis size and shape using sets of measurements that are independent of jaw size, position, inclination, and age. Assuming that the “sexual selection” theory is defensible, we would expect the following: (1) sexual dimorphism will be more pronounced in the chin than in the symphysis, and (2) chin size and shape will be more variable than symphysis size and shape.

## 2. Materials and Methods

### 2.1. Study Sample

The studied population included 419 adults of Caucasian origin. The minimum number of individuals was calculated using WinPepi software (v.11.65, J.H. Abramson, USA). It required at least 175 individuals per group (males and females) (total *n* = 350) to provide a significant difference with a 0.3 effect size for *p* < 0.05 and power = 80%. The included individuals had undergone a head and neck CT scan (Brilliance 64, Philips Medical System, Cleveland, OH, USA) for medical diagnostic purposes (unrelated to the present study) at Carmel Medical Center, Haifa, Israel. The following parameters were used: slice thickness 0.9–3.0 mm, pixel spacing 0.3–0.5 mm, 120 kV, 250–500 mAs, the number of slices 150–950, and matrix 512 × 512.

The inclusion criteria were as follows: age ≥ 18 years, intact lower incisors, teeth at the centric occlusion (maximum intercuspation), and the presence of at least two teeth of the posterior unit (premolars and/or molars) on each side. The exclusion criteria were as follows: (1) the absence of lower incisors; (2) dental implants and metal restorations that impede measurements; (3) evidence of orthodontic treatment (e.g., brackets, appliances, and lingual fixed retainers); (4) previous surgery in the head and neck region (based on medical files or signs on the skull); (5) prominent facial and mandibular asymmetry, cranio-facial, temporo-mandibular joint, and muscle disorders; (6) trauma; and (7) technically aberrant CT scans. The research was approved by the ethics committee of the Carmel Medical Center (CMC 11-0066).

### 2.2. Measuring the Chin and Symphysis

**Figure 2:**
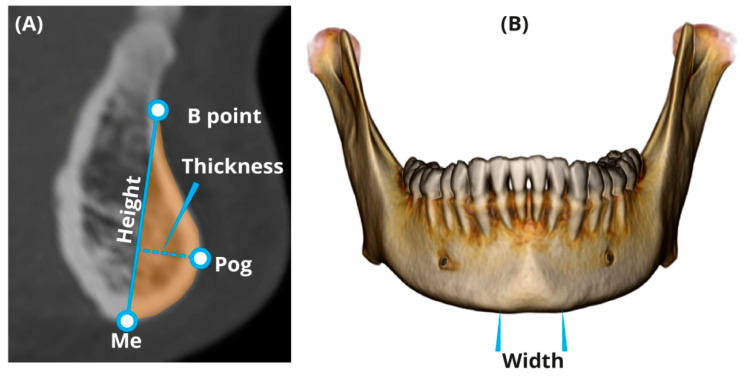
Measurements of the chin. (**A**) Height, thickness, and cross-sectional area (CSA) (in light orange); Me—menton, Pog—pogonion. (**B**) Chin width.

**Figure 3:**
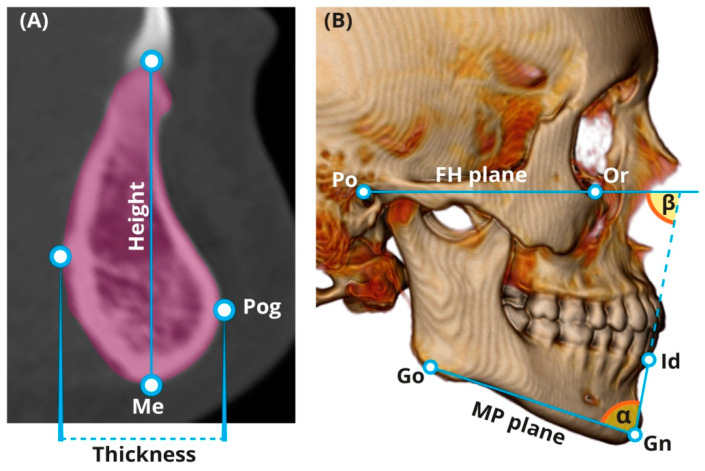
Measurements of symphysis. (**A**) Height, thickness, and CSA (in light pink). (**B**) Symphysis orientation (β angle) and inclination (α angle). Me—menton, Pog—pogonion, Po—porion, Or—orbitale, Go—gonion, Id—infradentale, Gn—gnathion, MP—mandibular plane, and FH—Frankfort horizontal.

Before measuring the chin and symphysis, a standardized alignment of the skull parallel to the Frankfort horizontal (FH) plane was performed. Landmarks of the chin and symphysis were identified according to Caufield [25] and Swennenet al. [26]. All measurements were taken directly from the CT scans using a multi-planner reformatting technique (“Extended Brilliance Workspace” portal (v2.6.0.27) (Philips Medical Systems, Cleveland, OH, USA). A set of four parameters (linear and cross-sectional area (CSA)) was developed to evaluate the chin size and shape, and five parameters were developed for evaluating the symphysis, including angulations (Table 1). Most parameters of the chin and symphysis were measured at the midsagittal section (Figure 2A and Figure 3). Chin width was measured using the volume-rendering model from a frontal view (Figure 2B). All of the CSAs were extracted from the CT software using manual tracing of the anatomical borders of the structure. The reliability of the measurements was assessed using 15 randomly selected CT scans of the head and neck. To evaluate the intra-observer variation, a single researcher (T.S.T.) carried out the measurements twice with a two-week interval between each attempt. The mean of the two measurement sets carried out for reliability assessment was utilized for further statistical analysis.To evaluate the inter-observer variation, the measurements were carried out by an additional independent researcher (H.M.).

Additionally, three indices were calculated: chin size index (%)—the ratio between the chin CSA and the symphysis CSA, multiplied by 100; chin shape index (%)—the ratio between the chin thickness and the chin height, multiplied by 100; and symphysis shape index (%)—the ratio between the symphysis thickness and the symphysis height, multiplied by 100.

### 2.3. Size Factor

All linear and CSA measurements were controlled for general mandibular size, as expressed by its geometric mean (MGM—mandibular geometric mean). The latter was calculated based on a set of six linear measurements: ramus length and width, mandibular body length, gonial width, coronoid length, andbigonial breadth, following the methods described by Sella Tunis et al. [28]. All measurements were taken directly from the CT scans using the Brilliance Workspace Portal (Philips v. 2.6.1.5, Philips, Amsterdam, Netherlands). The square roots of the chin and symphysis CSAs were divided by the MGM, following the principles presented in Jungers et al. [29].

### 2.4. Age Factor

Since the size and shape of the mandible are very dynamic [30] and may change considerably with age, there was a need to locate measures that are age-dependent. Possible associations between the individual’s chronological age and all chin and symphysis parameters were assessed using different types of correlation analysis. Moreover, since different age distributions for males and females can easily bias the results, we ensured that the samples of males and females were age-matched.

### 2.5. Orientation Factor

Previous studies suggested a possible association between chin morphometrics and the mandibular plane angle (MPa) [31,32]. Therefore, in searching for possible associations between chin morphometrics and other mandibular parameters, one needs to control for the MPa to avoid a potential confounder effect. This is especially important when looking for an association between the chin and the symphysis metrical characteristics and age since the MPa is age-dependent [33,34]. Initially, our measuring methods of the chin and symphysis were constructed to be independent of MPa, i.e., the head was positioned parallel to the FH plane and all of the landmarks and lines were drawn independent of the MP. Additionally, to control for the MPa, we used partial correlation analysis when searching for associations between the chin and symphysis parameters and age. The MPa was measured following Downs’ [35] method, relative to the FH plane.

### 2.6. Statistical Analysis

The data were recorded and analyzed using IBM SPSS statistics, version 20 software (IBM Corp., Armonk, NY, USA). The level of statistical significance was set at *p* < 0.05. The intraclass correlation coefficient (ICC) was calculated to examine the reproducibility of the measurements; It was interpreted according to the categorization method of Cicchetti [36]. One-sample Kolmogorov–Smirnov tests and histograms were carried out to determine the normality of the distributions of the measurements. Descriptive statistics (mean, SD, and range) were carried out for all parameters. The coefficient of variation (CV) was defined as the standard deviation expressed as a percentage of the mean; it was calculated for each sex separately to estimate the extent of the chin and symphysis variation. The F-statistic and *p*-values were calculated to compare the chin and symphysis CVs between the sexes according to Forkman [37] using MedCalc statistical software (version 19.3.1, MedCalc Software bv, Ostend, Belgium; https://www.medcalc.org; 2016)).

The CV was classified according to the system devisedby Vazet al. [38]. Independent-sample *t*-tests (two-tailed) were carried out to detect significant differences in the observed values of the chin and symphysis between males and females, and to examine differences in the mean age between the sexes. The Mann–Whitney test was carried out to detect significant differences in the controlled values of the chin and symphysis and their indexes between the sexes. Pearson correlations were calculated to test for associations between the chin and symphysis size and age. A partial correlation was calculated between the chin and symphysis parameters and age, while controlling for the MPa, to eliminate its potential role as a confounder. Linear regression analysis was carried out to evaluate the percentage of the chin and symphysis variance that was accounted for by sex and size. 

## 3. Results

### 3.1. Reliability Analysis

The intra-observer variation of all measurements showed excellent results (0.838 ≤ ICC≤ 0.986) and the inter-observer variation showed good to excellent results (0.704 ≤ ICC ≤ 0.980) (*p* < 0.001).

### 3.2. Age Factor

The population studied included 419 adults: 203 males (48.4%) and 216 females (51.6%) between 18–96 years of age (Table 2). Males and females were age-matched, i.e., no significant difference was found in the mean age between males and females (*p* = 0.052). Age was normally distributed in both males and females (Kolmogorov–Smirnov test: *p* = 0.076 and *p* = 0.143, respectively).

In both sexes, no significant associations were found between the chin parameters (observed values) and age, except for a very weak association in males for chin width (r = 0.146) (Table 3). Symphysis height was significantly negatively associated with age in both males (r = −0.371) and females (r = −0.268), and its shape index was significantly positively associated with age (males r = 0.366, females r = 0.275). Symphysis thickness and CSA were independent of age (*p* > 0.093). Symphysis inclination (relative to the MP) did not vary with age, regardless of sex (*p* > 0.315), although its orientation relative to the FH had a significant yet weak positive correlation with age (r = 0.247 and r = 0.173 in males and females, respectively). We found a significant correlation between chin width and the MPa (males r= −0.243, *p* < 0.001; females r = −0.416, *p* < 0.001). Regarding its potential confounder effect, a partial correlation was calculated (controlling for the MPa). No significant associations between the chin parameters and age were found after controlling for the MPa.

### 3.3. Chin Morphometrics in Males and Females

In the observed values, chin height and chin width were significantly greater in males than in females, although chin height was slightly greater in males (mean 21.6 mm vs. 21.0 mm in females). No significant difference was observed in chin thickness and CSA between the sexes. Chin height and the CSA were significantly greater in females than in males when controlling for the MGM (Table 4). Similar to the observed values, the controlled values for chin thickness did not differ significantly between the sexes and the chin width exhibited significantly greater values in males. No significant difference was found in the chin shape index between males and females; however, the size index was significantly greater in females (i.e., in females, the chin comprised 17.9% of the symphysis CSA; in males, it was 16.5% (Table 4)). The CVs of the studied parameters ranged from 12.3–35%, the low to high rates, respectively, according to the system devisedby Vazet al. [38] (Table 5). The parameter manifesting the highest variation was the chin CSA and the one exhibiting the lowest variation was the chin height. The CV was independent of sex in four of the six traits studied, whereas the CV was significantly greater in females in the two other traits (chin width and shape index).

### 3.4. Symphysis Morphometrics in Males and Females

In the observed values, males exhibited a significantly higher, thicker, and larger symphysis CSA compared with females (Table 6). However, no significant difference between the sexes was found when the symphysis height, thickness, and CSA were controlled for mandibular size (*p* > 0.085). No significant difference in the symphysis shape index was found between the sexes: the symphysis-thickness-to-symphysis-height ratio was approximately 1:2 (Table 6). In contrast, the symphysis orientation was significantly greater in males (80.24°) than in females (78.30°) (*p* = 0.011), and the symphysis inclination was sex-independent (*p* = 0.905). This implied that males’ symphyses were more lingually oriented than females’ symphyses. The overall results suggested that symphysis size (the observed values) was significantly greater in males than in females. Nevertheless, when controlling for the MGM, no significant differences existed in the symphysis morphometrics between the sexes. Additionally, the symphysis shape was similar between males and females. The CV for all symphysis parameters was found to be low in both sexes, ranging between 7.2% (symphysis inclination) and 17.4% (symphysis CSA) (Table 7). Nevertheless, males exhibited a statistically significantly greater variation in symphysis height and thickness compared with females (*p* < 0.044). No significant differences between the sexes were observed for the CV of the symphysis CSA, shape index, orientation, and inclination. 

### 3.5. Comparing Chin and Symphysis Variations

In both sexes, the CVs for the symphysis height, thickness, and CSA were found to be significantly lower than the CVs for the corresponding parameters in the chin (*p* < 0.001) (Table 8). 

### 3.6. Size and Sex Factors 

After evaluating the results presented in Table 9, it was clear that mandible size was responsible for a greater portion of the explained variance than sex. Regarding the chin parameters, the mandible size accounted for a very small percentage of the variance, although it was statistically significant (Table 9), ranging between 2% (chin width) and 14% (chin height) of the variance. In the symphysis, a much larger portion was explained by mandible size, ranging between 24% (symphysis thickness) and 33% (symphysis CSA). Sex accounted for only a minute portion of the variance (1–4%) in both the chin and symphysis parameters, excluding the chin width (16%).

## 4. Discussion

### 4.1. Sex Differences in Chin Size and Shape

The present study showed that males had a significantly wider and taller chin than females, a finding that is in accordance with previous findings [15,16,17,18]. Nevertheless, when these measures of the chin were controlled for mandibular size, the opposite was observed for chin height, i.e., the female chin was relatively higher than the male chin. Our findings contradict previous studies suggesting that males display a more prominent mental eminence than females [9,17]. According to the current study, the chin thickness did not differ significantly between the sexes, either regarding the observed values or the controlled values for mandibular size. This is in accordance with the findings of Daegling [23], who did not find any difference between the sexes regarding chin thickness, shape, and size. Additionally, a recent study by Pampush [24] did not reveal any sex differences in chin measures in the midsagittal plane. However, as previously mentioned, Thayer and Dobson [9] compared the chin shape between the sexes and found that sexual dimorphism exists in its shape. This discrepancy between the studies can probably be attributed to the methodology applied to evaluate the shape and size of the symphysis and chin. Additionally, Thayer and Dobson [9] did not control their measurements for mandibular size. 

### 4.2. Sex Differences in Symphysis Size and Shape

Our findings clearly show the existence of sexual dimorphism in the observed symphysis metric characteristics, i.e., males exhibit higher, thicker, and larger symphyses that are more lingually oriented compared with females. However, we found that males and females possess similar symphyseal shapes and similar sizes after controlling for mandibular size. Swastyet al. [39] reported higher and thicker symphyses in males; however, they did not control for mandibular size. Similarly, Aki et al. [40] reported higher and deeper symphyses in males than in females; the ratio between the two (height/depth) was slightly greater in females. Since no difference between the sexes was found in the symphysis inclination relative to the MP, we suggest that the male symphysis was more lingually oriented relative to the FH plane due to a smaller mandibular angle, a finding already reported by us [41].

### 4.3. Age Influence on Chin and Symphysis

A previous study suggested that the magnitude of sexual dimorphism in human’s mandibular size and shape is age-dependent [20]. Although our study was retrospective, from the obtained data correlated with age, it became clear that chin features are age-independent.

Similar findings were reported by Haskell [42]. In contrast to chin height, which remains stable from the age of 18 years onward, symphysis height decreases with age. The decrease in the symphysis height can explain the increase of its shape index, which leads to the development of a more square-shaped symphysis with age in both sexes. An age-related reduction in alveolar bone crest height was reported in the literature; this is usually attributed to deterioration in the periodontal status or to tooth loss with age [43,44]. However, a reduction in alveolar bone crest height with age was also found in individuals with healthy dental and periodontal status; mandibular incisors displayed the greatest alveolar bone loss [45].

**Figure 4:**
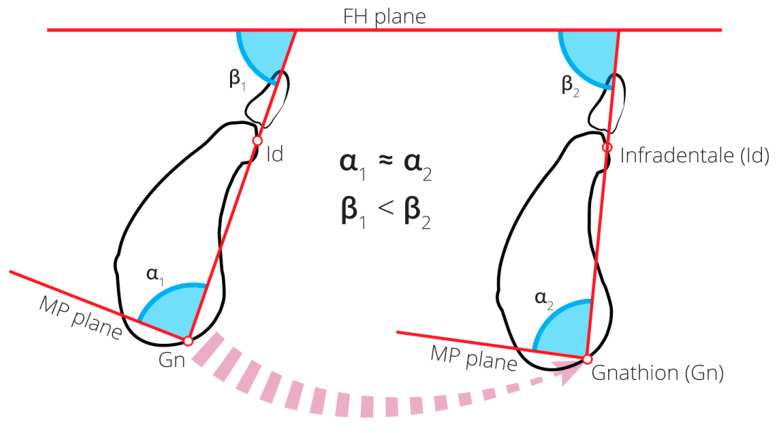
Changes in the symphysis orientation and inclination with age. The illustration on the left represents younger individuals, whereas the one on the right represents older individuals. No change in the symphysis inclination relative to the MP (α angle) was evident (α_1_ is similar to α_2_), whereas the symphysis orientation relative to the FH plane (β angle) increased with age (β_1_ < β_2_); this was probably due to a forward rotation of the mandible (arrow).

According to our study, symphysis orientation (measured relative to FH) changed significantly with age. This implies that with age, the symphysis became more lingually oriented (retro lined) in both males and females. Since no significant change in symphysis inclination (measured relative to the MP) with age was evident, we hypothesized that the change in the symphysis orientation with age was mainly due to a continuous forward rotation of the mandible over time (Figure 4). A reduction in crown height (due to attrition/abrasion) and/or posterior tooth loss with age might lead to anterior rotation of the mandible, and subsequently, may lead to a reduction in the MPa and to a lower anterior facial height [46]. Age changes in the adult facial profile were studied by Formby et al. [33], who found a decrease in the MPa relative to the cranial base with age in males. They attributed this phenomenon to the mandibular forward rotation with age. In a longitudinal study, Pecora et al. [34] found differences between males and females regarding the degree of mandibular rotation with age: the mandibles of females undergo posterior rotation, whereas those of males undergo anterior rotation. This rotation of the mandible is followed by a relative protrusion of the chin.

### 4.4. Evolutionary Implication: “Sexual Selection”

Based on the notion of its sex-specific characteristics, it has been suggested that the human chin results from long-term sexual selection [8,9,16,47]. Facial appearance is extensively influenced by the size and shape of the chin [10,48]. An interesting finding of our study relates to the sexual dimorphism in the size and shape of the chin. Contrary to the common notion that chin thickness is more pronounced in males, our findings suggest that no significant differences in chin thickness (observed and controlled) and chin shape existed between the sexes. Although the observed chin height dimension was greater in males, when controlling for mandibular size, females possessed relatively higher chins. Moreover, when controlling for mandibular size, the chin CSA showed a greater dimension in females than in males, and its size index (chin CSA/symphysis CSA) was smaller in males compared with females, indicating that the chin was, in fact, smaller in males than in females. Only chin width (the frontal aspect) was considerably greater in males than in females in both the observed and controlled measures.

In the chin, except for chin width, mandibular size accounted for 6 to14% of the variance (it varied between the measures) and sex accounted for 1–2%. However, this phenomenon (variance explained by size > variance explained by sex) was reversed regarding chin width, where sex accounted for 16% of the variance and size for only2%. Notably, the chin width CV rate significantly differed between males and females, i.e., males showed significantly lower variation rates (CV) compared with females, suggesting a stronger selection. In the symphysis, mandibular size accounted for 24–33% of the variances of various traits, sex contributed only 4% to the height dimension, and null to thickness and CSA.

The symphysis only showed a pronounced sexual dimorphism in its observed values. Males exhibited a greater symphysis size than did females (height, thickness, and larger CSA). However, no sexual dimorphism existed after controlling for mandibular size. This suggests that the differences between the sexes could only be attributed to the greater and more robust mandibles of males. Additionally, the symphysis shape did not differ between males and females. As mentioned before, between 24–33% of the symphysis variance was explained by mandibular size, whereas only up to 4% was explained by sex. 

The above findings lend only partial support to the “sexual selection” theory, suggesting that other factors may also be involved in chin formation. Therefore, after studying the chin size and shape, we concluded that the possible input of other factors besides sexual selection should be considered. Future studies are needed to reveal the magnitude and direction of chin and symphysis sexual dimorphism, as well as the role of facial attractiveness in their expression.

## 5. Study Limitations

This is a retrospective study. The findings of the current study were based on a population of Caucasian origin. A generalization of the results requires further study of the different populations of different geographical regions. Information on previous orthodontic treatment was unavailable. However, to avoid a possible confounding effect of the orthodontic treatment on the chin and symphysis size and shape, individuals who showed indirect evidence of such treatment were excluded from the study.

## 6. Conclusions

Chin width (the frontal view) was found to be a sexually selected trait; it can be considered as a parameter for sex determination (males possess significantly wider chins than females do). Chin thickness (the lateral view) is similar in both sexes. The symphysis was size-dependent and its size and shape were sex-independent. The chin was found to be a more heterogeneous anatomical structure than symphysis and it was sexually more dimorphic.

## Figures and Tables

**Table 1:** Definitions of the chin and the symphysis measurements.

Measurement		Definition
Chin	Height (mm)	The distance between the most posterior midline point in the concavity of the mandibular symphysis (B point) and the menton
	Thickness(mm)	The maximum thickness of the chin, measured as the length of the perpendicular line from pogonion to the chin height line
	CSA (mm^2^)	The portion of the symphysis CSA that is located anterior to the chin height line
	Width (mm)	The distance between the right and left mental tubercles
Symphysis	Height (mm)	The distance between the most superior point on the alveolar bone and menton
	Thickness (mm)	The distance between the pogonion and the most posterior point on the symphysis
	CSA (mm^2^)	The total CSA of the symphysis in the midsagittal plane
	Inclination (°)	Inclination of the symphysis relative to the MP: the angle (α angle) created between the line passing from the infradentale (the midline point at the superior tip of the septum between the mandibular central incisors) to the gnathion (Id-Gn line), and the line passing from gonion to gnathion [27]
	Orientation (°)	Inclination of the symphysis relative to the FH plane: the angle (β angle) measured at the cross-point between the Id-Gn line and the FH plane (the porion-orbitale line)

Note: CSA- cross-sectional area; MP-mandibular plane; Id-infradentale; Gn-gnathion; FH- Frankfort horizontal.

**Table 2:** Descriptive statistics for chronological age (in years) by sex.

Statistics	Males	Females	Total
Mean	51.46	55.39	53.48
SD	20.357	20.823	20.668
Minimum	18	18	18
Maximum	96	96	96
Range	78	78	78
*n*	203	216	216

Note: SD—standard deviation.

**Table 3:** Correlation coefficients between the chin and symphysis parameters and age by sex (males *n* = 203, females *n* = 216).

Measurement		Sex	Observed		Controlled for the MPa ^b^	
			r	*p*-Value ^a^	r	*p*-Value ^a^
Chin	Height	Male	−0.077	0.275	−0.062	0.408
		Female	0.008	0.912	−0.010	0.894
	Thickness	Male	0.061	0.387	0.035	0.637
		Female	−0.025	0.717	−0.083	0.246
	CSA	Male	0.062	0.380	0.027	0.722
		Female	0.022	0.748	−0.018	0.796
	Width	Male	0.146	**0.038**	0.110	0.140
		Female	0.094	0.170	0.048	0.497
	Shape index	Male	0.101	0.151	0.072	0.334
		Female	−0.022	0.748	−0.103	0.147
	Size index	Male	0.120	0.089	0.037	0.619
		Female	0.048	0.486	0.009	0.901
Symphysis	Height	Male	−0.371	**<0.001**	−0.282	**<0.001**
		Female	−0.268	**<0.001**	−0.237	**0.001**
	Thickness	Male	0.118	0.093	0.118	0.112
		Female	0.083	0.225	0.052	0.464
	CSA	Male	−0.111	0.114	−0.012	0.877
		Female	−0.023	0.737	−0.027	0.701
	Shape index	Male	0.366	**<0.001**	0.311	**<0.001**
		Female	0.275	**<0.001**	0.246	**<0.001**
	Orientation	Male	0.247	**0.001**	0.169	**0.023**
		Female	0.173	**0.013**	0.166	**0.019**
	Inclination	Male	0.027	0.713	−0.019	0.798
		Female	−0.071	0.315	−0.086	0.228

Note: ^a^ Significant *p*-values (*p* < 0.05) are denoted in bold; ^b^ Partial correlations between chin and symphysis parameters and age, controlling for the MPa. CSA-cross-sectional area.

**Table 4:** Morphometric characteristics of the male and female chin (males *n* = 203, females *n* = 216).

Chin Measurement	Sex	Mean	SD	Minimum	Maximum	*p*-Values *	
						Observed	Controlled
						Measures	Measures
Height (mm)	Male	21.58	3.102	13.30	28.900	0.046	<0.001(F > M)
	Female	21.02	2.576	14.60	26.400		
Thickness (mm)	Male	4.00	0.991	1.40	7.100	0.176	0.079
	Female	3.86	1.054	1.00	6.800		
CSA (mm^2^)	Male	53.04	18.534	13.80	113.400	0.120	0.001(F > M)
	Female	50.32	17.181	11.70	110.500		
Width (mm)	Male	28.18	5.622	16.30	42.700	<0.001	<0.001(M > F)
	Female	23.18	5.712	10.80	43.800		
Shape index (%)	Male	18.58	3.874	5.32	28.571	0.565	
	Female	18.34	4.285	4.55	32.850		
Size index (%)	Male	16.50	5.550	5.29	33.744	0.010	
	Female	17.88	5.797	4.79	34.043		

Note: * Significant *p*-values (*p* < 0.05) are denoted in bold; *p*-values are presented for both the observed andthe controlled (MGM) measures when they are compared between the sexes; SD—standard deviation; M—males; F—females; CSA-cross-sectional area.

**Table 5:** Variation in chin morphometrics in males and females (males *n* = 203; females *n* = 216).

Chin Measurement	Sex	CV	F-Statistic	*p*-Values ^a^	CV Classification ^b^
Height	Male	14.374	0.731	0.024	Low
	Female	12.257			Low
Thickness	Male	24.779	1.198	0.193	High
	Female	27.293			High
CSA	Male	34.945	0.959	0.764	Medium
	Female	34.144			Medium
Width	Male	19.952	1.495	0.004	Low
	Female	24.640			Medium
Shape index	Male	20.848	1.243	0.118	Medium
	Female	23.368			High
Size index	Male	33.635	0.936	0.631	High
	Female	32.419			High

Note: ^a^ Significant*p*-values (*p* < 0.05) are denoted in bold; ^b^ Classification was according to the system devisedby Vazet al. [38] for variables with a normal distribution. CSA-cross-sectional area; CV—Coefficient of variation.

**Table 6:** Morphometric characteristics of the male and female symphysis (males *n* = 203, females *n* = 216).

Symphysis Measurement	Sex	Mean	SD	Minimum	Maximum	*p*-Values *	
						Observed	Controlled
						Measures	Measures
Height (mm)	Male	33.28	3.303	24.60	42.600	<0.001	0.087
	Female	30.10	2.581	23.40	36.500		
Thickness (mm)	Male	15.46	2.050	11.20	23.200	<0.001	0.403
	Female	14.36	1.652	10.40	20.200		
CSA (mm^2^)	Male	324.72	56.530	186.70	481.100	<0.001	0.085
	Female	283.77	45.244	189.20	414.100		
Shape index (%)	Male	46.78	7.036	32.37	70.732	0.073	
	Female	47.96	6.302	31.95	66.096		
Orientation (°)	Male	80.24	7.738	57.10	103.000	0.011	
	Female	78.30	7.300	52.90	98.800		
Inclination (°)	Male	75.82	5.487	61.00	89.000	0.905	
	Female	75.75	5.861	56.00	92.000		

Note: * Significant *p*-values ( *p*< 0.05) are denoted in bold; *p*-values are presented for both the observed and the controlled (MGM) measures when they are compared between the sexes; SD—standard deviation; CSA-cross-sectional area.

**Table 7:** Variation in symphysis morphometrics in males and females (males *n*=203, females *n*=216).

Symphysis Measurement	Sex	CV	F-Statistic	*p*-Values ^a^	CV Classification ^b^
Height	Male	9.924	0.748	0.036	Low
	Female	8.574			Low
Thickness	Male	13.262	0.756	0.044	Low
	Female	11.510			Low
CSA	Male	17.409	0.843	0.217	Low
	Female	15.944			Low
Shape index	Male	15.040	0.767	0.056	Low
	Female	13.141			Low
Orientation	Male	9.644	0.935	0.639	Low
	Female	9.323			Low
Inclination	Male	7.237	1.142	0.362	Low
	Female	7.737			Low

Note: ^a^ Significant*p*-values (*p* < 0.05) are denoted in bold; ^b^ Classification was according to the system devised byVazet al. [38] for variables with a normal distribution. CV—Coefficient of variation; CSA-cross-sectional area.

**Table 8:** Variation comparison of the chin and symphysis parameters in males and females (males *n* = 203, females *n* = 216).

Chin and Symphysis Measurements ^a^	Sex	F-Statistic	*p*-Values *
Height	Male	0.482	<0.001
	Female	0.493	<0.001
Thickness	Male	0.299	<0.001
	Female	0.189	<0.001
CSA	Male	0.270	<0.001
	Female	0.237	<0.001

Note: ^a^ F-statistic and *p*-values for comparison of the chin and symphysis CVs between the sexes; * Significant *p*-values (*p* < 0.05) are denoted in bold. CSA-cross-sectional area.

**Table 9:** The effect of sex and size on the chin and symphysis (males *n*=203, females *n*=216).

Dependent Variable		Independent Variables				Model Summary ^#^	
		Sex		Size (MGM)		R^2^	*p*-Value *
		R^2 a^	*p*-Value *^,b^	R^2 a^	*p*-Value *^,b^		
Chin	Thickness	0.01	0.034	0.06	<0.001	0.07	<0.001
	CSA	0.02	0.003	0.10	<0.001	0.12	<0.001
	Height	0.02	0.001	0.14	<0.001	0.16	<0.001
	Width	0.16	<0.001	0.02	0.005	0.18	<0.001
Symphysis	Thickness	0.00	0.879	0.24	<0.001	0.24	<0.001
	Height	0.04	<0.001	0.27	<0.001	0.31	<0.001
	CSA	0.00	0.299	0.33	<0.001	0.33	<0.001

Note: ^#^ Linear regression forward method; ^a^ R-squared (R^2^) change; ^b^ *p*-value change; * Significant *p*-values (*p* < 0.05) are denoted in bold. CSA-cross-sectional area; MGM- mandibular geometric mean.
